# A Neurophysiologically Plausible Population Code Model for Feature Integration Explains Visual Crowding

**DOI:** 10.1371/journal.pcbi.1000646

**Published:** 2010-01-22

**Authors:** Ronald van den Berg, Jos B. T. M. Roerdink, Frans W. Cornelissen

**Affiliations:** 1 Institute of Mathematics and Computing Science, University of Groningen, Groningen, The Netherlands; 2 Laboratory for Experimental Ophthalmology, University Medical Center Groningen, University of Groningen, Groningen, The Netherlands; 3 School for Behavioral and Cognitive Neurosciences, University of Groningen, Groningen, The Netherlands; Northwestern University, United States of America

## Abstract

An object in the peripheral visual field is more difficult to recognize when surrounded by other objects. This phenomenon is called “crowding”. Crowding places a fundamental constraint on human vision that limits performance on numerous tasks. It has been suggested that crowding results from spatial feature integration necessary for object recognition. However, in the absence of convincing models, this theory has remained controversial. Here, we present a quantitative and physiologically plausible model for spatial integration of orientation signals, based on the principles of population coding. Using simulations, we demonstrate that this model coherently accounts for fundamental properties of crowding, including critical spacing, “compulsory averaging”, and a foveal-peripheral anisotropy. Moreover, we show that the model predicts increased responses to correlated visual stimuli. Altogether, these results suggest that crowding has little immediate bearing on object recognition but is a by-product of a general, elementary integration mechanism in early vision aimed at improving signal quality.

## Author Summary

Visual crowding refers to the phenomenon that objects become more difficult to recognize when other objects surround them. Recently there has been an explosion of studies on crowding, driven, in part, by the belief that understanding crowding will help to understand a range of visual behaviours, including object recognition, visual search, reading, and texture recognition. Given the long-standing interest in the topic and its relevance for a wide range of research fields, it is quite surprising that after nearly a century of research the mechanisms underlying crowding are still as poorly understood as they are today. A nearly complete lack of quantitative models seems to be one of the main reasons for this. Here, we present a mathematical, biologically motivated model of feature integration at the level of neuron populations. Using simulations, we demonstrate that several fundamental properties of the crowding effect can be explained as the by-product of an integration mechanism that may have a function in contour integration. Altogether, these results help differentiate between earlier theories about both the neural and functional origin of crowding.

## Introduction

Since Korte [1] originally described perceptual phenomena of reading in peripheral vision, a substantial number of studies have shown the important role of spacing for object recognition. The phenomenon that an object becomes more difficult to recognize when surrounded by other objects is now popularly known as ‘crowding’ [2] (see [3],[4] for two recent reviews).

**Figure 1:**
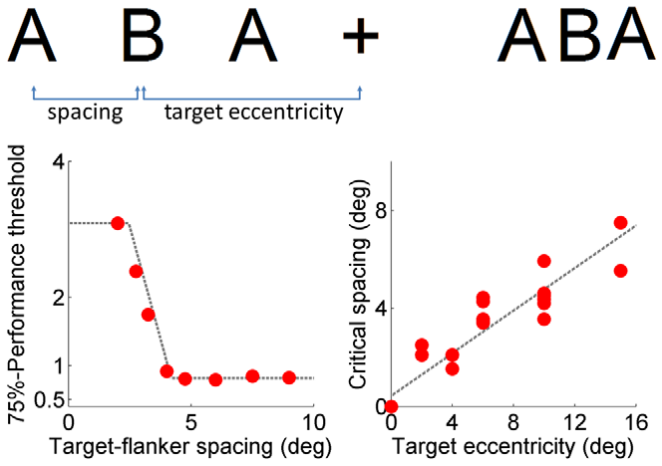
An example demonstrating the crowding phenomenon. Top: The two B's are at equal distance from the fixation cross. On the left, where the center-to-center spacing between the letters is approximately one half of the eccentricity of the central letter, the “B” can easily be recognized when fixating the cross. Letter spacing on the right is much smaller, and the “B” appears to be jumbled with its neighbors. Bottom, left: Human data from a typical crowding experiment. Crowding diminishes as target-flanker spacing is increased, up to a certain critical spacing after which flankers have no effect. Bottom, right: Findings from psychophysical studies show that critical spacing is a linear function of target eccentricity. Data from [12].

The strength of the crowding effect depends on the spacing between objects (Figure 1). The largest spacing at which there is a measurable effect is commonly referred to as the ‘critical spacing’. An important and often replicated finding is that the critical spacing for object recognition is proportional to the viewing eccentricity [5]. Moreover, critical spacing is found to be highly invariant to a great variety of stimulus manipulations, such as contrast and size [6]–[8]. Critical spacing is the most extensively studied crowding property and, because of its robustness, now sometimes considered the defining property of crowding [3].

Crowding is a general phenomenon in vision. It is not confined to letter and shape recognition, but affects a broad range of stimuli and tasks, including the identification of orientation [9]–[11], object size, hue and saturation of colors [12], recognition of faces [13],[14], reading [15], and visual search [16]–[18]. Altogether, crowding emerges as a fundamental limiting factor in vision, making the question about its neural basis and functional origin rather pressing.

Several theories have been proposed to explain the crowding effect [4],[19]. Currently, there is a growing consensus that crowding results from feature integration over an area that is larger than the target object [4]. However, there is a marked controversy about both the underlying mechanism and the functional origin of the effect. Some authors assert the existence of bottom-up hardwired integration fields (e.g., [3]), while others claim that feature integration arises from limitations related to the spatial resolution of attention (e.g. [20],[21]). Postulated functions of feature integration include texture perception [10], contour integration [22], and object recognition [3],[23]. In the absence of quantitative, biologically motivated models, however, it is not clear whether these theories can also quantitatively account for the ‘mysteries of crowding’ [4], and how plausible they are from a biological perspective.

Here, we present a quantitative model for spatial integration of orientation signals. Our model is based on the principles of population coding [24], which is an approach that mathematically formalizes the idea that information is encoded in the brain by populations of cells, rather than by single cells. Motivated by findings from physiological [25],[26] and theoretical [27] studies, we model feature integration as a (weighted) summation of population codes. Using simulations, we demonstrate that this approach allows to explain several fundamental crowding properties in a single, unified model, including aspects of critical spacing [6],[15], compulsory averaging of crowded orientation signals [10], and an asymmetry between the effects of foveally and peripherally placed flankers [28],[29]. Moreover, we show that the model predicts enhancement of signals that encode visual contours, which could facilitate subsequent contour detection and segmentation and adds support to earlier findings about a link between crowding and contour integration.

Altogether, our main finding is that feature integration, implemented in a neurophysiologically plausible way, produces crowding as a by-product. Furthermore, our results add support to an earlier suggested link between crowding and contour integration, and they point at V4 as a likely locus for feature integration cells (at least for the orientation domain).

## Results

### Model

Several different population coding schemes have been proposed in the literature [30]. Although they differ in their details, the general idea behind all of them is that variables are encoded in the brain by entire populations of cells. Our model is based on the ‘distributional population coding’ (DPC) scheme that was proposed by Zemel *et al.* [31]. In this scheme, a population code explicitly encodes a probability distribution over the stimulus domain. In this section we will only provide a general overview of our model. Mathematical details can be found in the Methods section.

**Figure 2:**
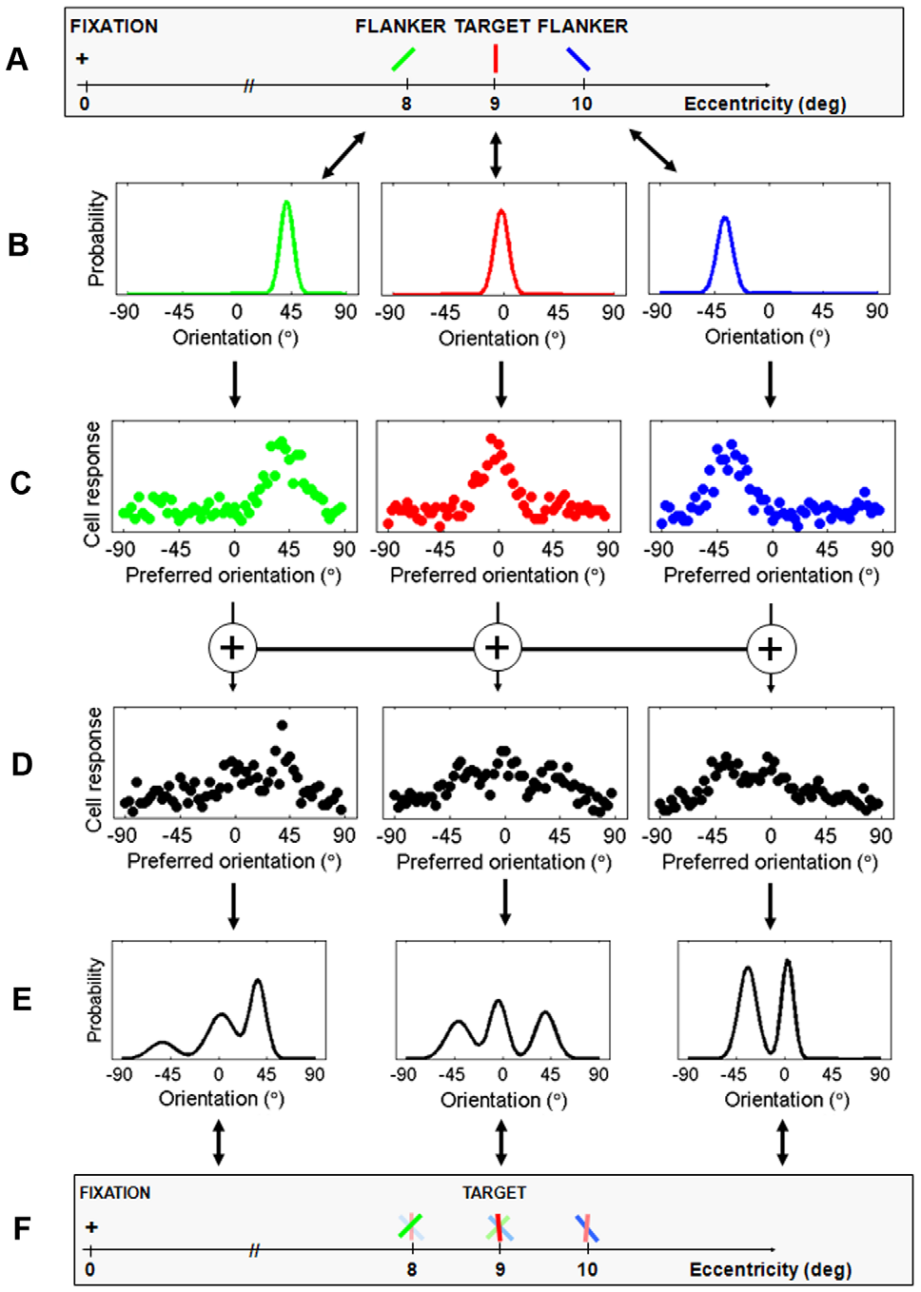
A graphical illustration of our model. A. In this example, the input consists of three oriented bars (the colors are only for visualization purposes and not part of the input to the model); B. Probability distributions are defined for the input stimuli; these distributions capture the stimulus uncertainty caused by neural noise in processing stages prior to the first layer of the model; C. In the first layer, a neural representation is computed for each of these distributions; D. In the second layer, the stimulus representation at each location is integrated with the representations of stimuli at neighboring locations. Integration is implemented as a weighted summation, such that nearby stimuli receive higher weights than stimuli that are far away; E. The resulting population codes are decoded to a mixture of normal distributions, with each component representing a perceived orientation at the respective location; F. Due to integration, the resulting percept of closely spaced stimuli will be crowded.

The input to the model consists of a set of stimuli, each one defined by a location, orientation, contrast, and size (Figure 2a). The first layer of the model represents full probability distributions over the input stimuli. These distributions are assumed to be Gaussian, with a width that depends on the eccentricity, contrast, and size of the stimuli (Figure 2b). Subsequently, these probability distributions are used as inputs to the DPC encoder that computes a population code representation for each of the stimuli (Figure 2c). The properties of the cells (e.g., tuning width) in the first layer are chosen such that they closely resemble V1 simple cells (see Methods for parameter values).

In the second layer, stimulus representations from the first layer are spatially integrated, in the form of weighted summations of cell responses (Figure 2d). The integration weights depend on the cortical distance in primary visual cortex between the locations of the ‘integration cell’ and the cells encoding the input stimuli (for details about the weight function and mapping of visual field to cortical locations, see Methods). This function can be interpreted as defining a cortical ‘integration field’. The size and shape of these integration fields can be thought of as representing the arborization of the dendritic tree, i.e., the distribution of lateral connections of a physiological integration cell. The weight function is a 2D Gaussian, thus reflecting that there are many short-range connections and fewer long-range connections. Unlike the first layer, which is a simulation of V1 simple cells, it is currently difficult to link the cells from the second layer to a very specific cortical area. Nonetheless, if we compare the predictions that follow from optimization of our model parameters to the current physiological literature, then we find V4 to be a likely candidate. We come back to this in the discussion section.

Several of the simulation experiments that we conducted required that a response be generated (e.g., when simulating psychophysical experiments involving target tilt estimation). In those simulations, a maximum-likelihood decoder was used to decode the post-integration population code associated with the target position back to a stimulus distribution (Figure 2e). The number of components of the returned mixture model was interpreted as the number of distinct orientations perceived at the location associated with the decoded population code, the mixing proportions as the amounts of evidence for the presence of an orientation, the means as estimates of these orientations, and the standard deviations as the amounts of uncertainty about these estimates.

### Critical regions for crowding

A well-established behavioral finding in human observers is that identification thresholds for a crowded target decrease as a function of target-flanker spacing until a certain critical spacing is reached. Beyond this critical spacing flankers no longer have an effect (see, for example, the results shown in Figure 1). In our model, the integration fields are implemented as weight functions of stimulus spacing in cortex. Consequently, flanker stimuli affect the identification of a target only when positioned within a certain distance from the target, yielding a critical region for target identification.

To examine whether our model can quantitatively account for critical regions found for human subjects, we performed a simulation that mimicked the psychophysical experiment by Pelli *et al.* [15], who estimated critical regions for letter identification at several positions in the visual field.

**Figure 3:**
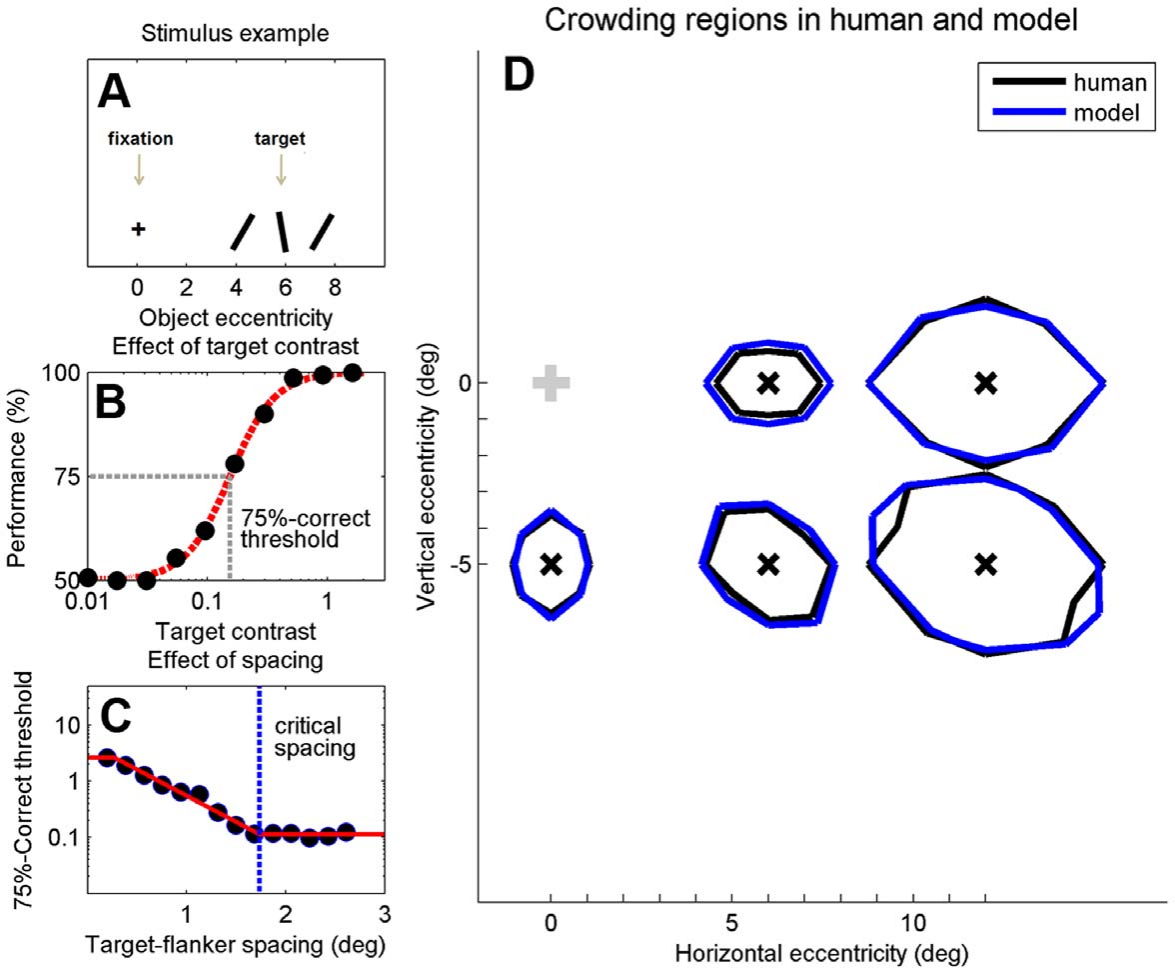
Comparison of crowding regions reported for humans with crowding regions estimated by our model. A. The input stimulus on each trial consisted of a ±10° tilted target stimulus and two 30° tilted flankers placed on opposite sides of the target. If the sign of the post-integration stimulus representation associated with the target position was the same as the sign of the input target, then performance on that trial was considered correct; B. Performance was estimated for a range of target contrasts, yielding a curve that is very similar to psychometric curves typically found with human experiments (compare, for example, with data shown in Figure 1). Based on these curves, contrast thresholds were estimated that produce 75% correct performance; C. Contrast thresholds decrease as target-flanker spacing is increased. The smallest spacing at which the flankers do not have an effect is defined as the critical spacing; D. Critical spacings were estimated in several directions around the target, at five different target positions. These simulation data accurately reproduce the critical regions measured psychophysically in humans. Human data from [15].

Critical regions predicted by our model were estimated as follows. For each target position, identification thresholds were determined for a range of target-flanker spacings (see Figures 3a and 3b; we refer to Methods for details about the procedure that was used to estimate identification thresholds). A ‘clipped line’ was fit to the resulting data, providing an estimate of the critical spacing (Figure 3c). By varying the positions of the flankers, we estimated critical spacing in several directions around the target. Combining these spacings gives an estimate of the critical region around a given target location (Figure 3d). We estimated model parameter values that result in a good model fit to one of the critical regions measured by Pelli *et al.* Subsequently, we repeated the experiment for the other target locations using the same parameter values, and found that the model accurately predicts all reported human critical regions (Figure 3d). These results thus provide quantitative evidence for the suggestion that the behavioral crowding regions found in humans can be explained as the result of fixed-sized, hard-wired integration fields in visual cortex.

### Effect of stimulus manipulations on critical spacing

The critical spacing for crowding is known to scale with eccentricity and is consistently found to be in the range 0.3–0.6 times the target eccentricity [6]. Moreover, it is found to be largely invariant under changes to the physical properties of the stimulus, such as the size, contrast, and number of flankers [6] and the ‘scaling’ of stimuli (i.e., changing the size of both the target and flankers) [6]–[8].

**Figure 4:**
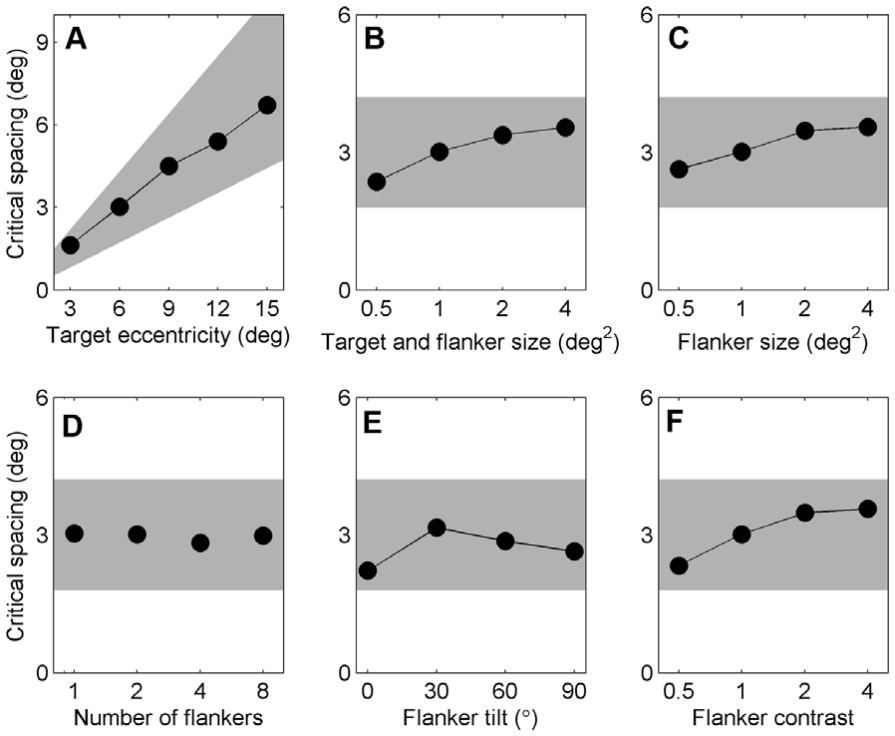
Simulation results showing the effect of several stimulus manipulations on estimated critical spacing. The shaded areas represent the range of critical spacings that are typically reported in the literature (0.3–0.6 times target eccentricity). Standard errors are smaller than the marker size. A. Critical spacing scales linearly with target eccentricity; B–F. Critical spacing is only weakly affected by various stimulus manipulations. The eccentricity of the target was 6 degrees in these experiments.

To further verify our model, we conducted another series of simulation experiments, in which we manipulated several stimulus properties. We found that the results are compatible with findings in human subjects: critical spacing predicted by our model scales linearly with target eccentricity and is hardly affected by stimulus manipulations (Figure 4).

### Compulsory averaging of crowded orientation signals

**Figure 5:**
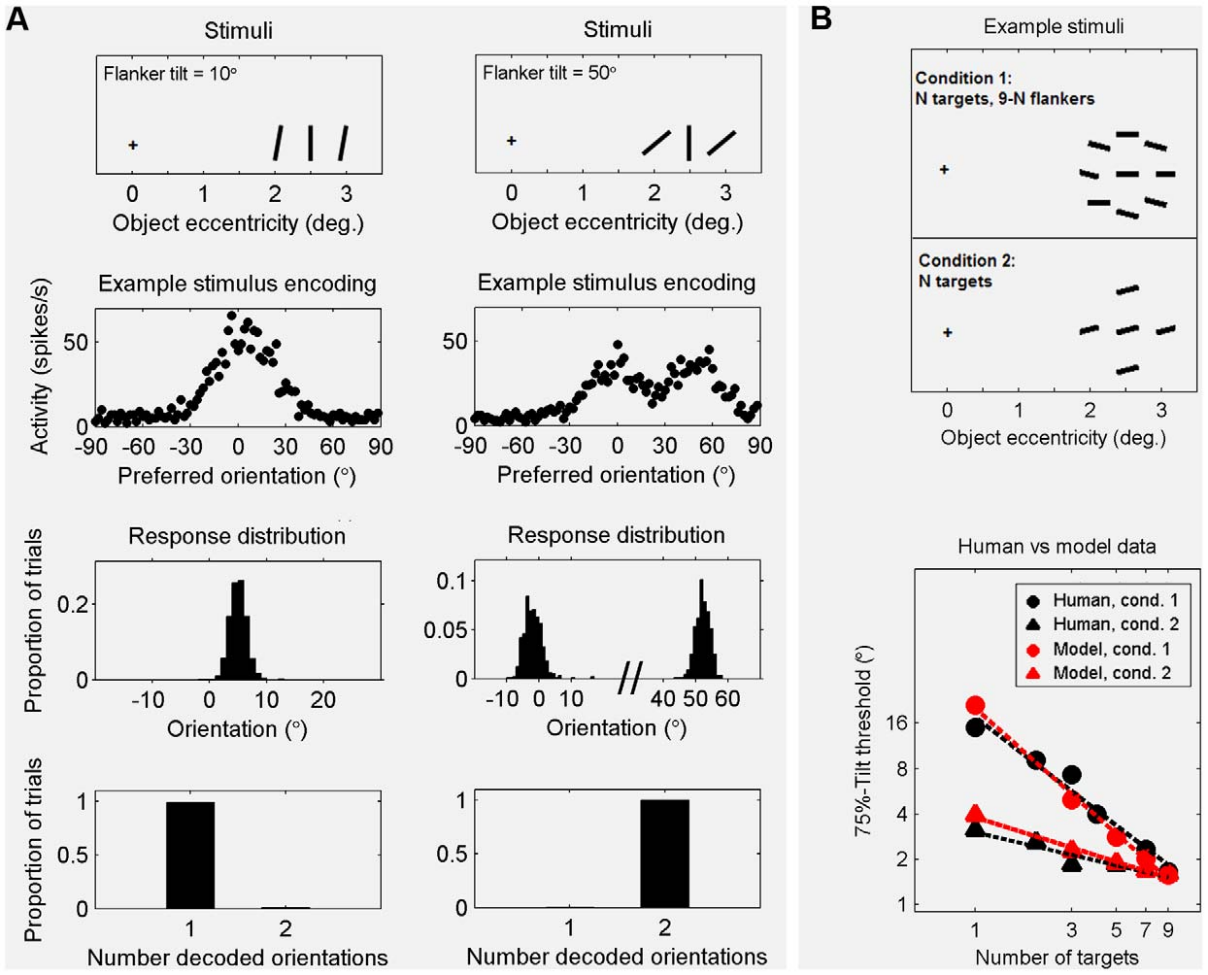
Compulsory averaging of crowded orientation signals explained as the result of ‘merging’ population codes. A. Simulation results illustrating how the ‘compulsory averaging’ effect arises in our model. Top row: example input stimuli, consisting of a vertical target flanked by two equally tilted flankers. Second row: single trial examples of population codes representing the post-integration stimulus at the target position. Third row: distributions of the orientations encoded at the target locations after integration (1000 trials). Bottom row: corresponding distributions of the number of perceived stimuli at the target position. When target and flanker tilt are nearly identical, their population code representations merge into a single hill of activity when integrated. The resulting code is decoded to a single orientation, with a value intermediate between the values of the input stimuli. This effect diminishes when the difference between target and flanker tilt is increased; B. Model fit to human psychophysical data. Top: Example stimuli of the experiment described in [10]. The task was to report the tilt direction of a variable number of equally tilted targets positioned within a set of horizontal flankers. Bottom: Identification thresholds predicted by our model are very close to those found for human subjects. Human data from [10], subject LP.

Human observers are able to report the mean orientation of a set of crowded stimuli, but not the orientations of the individual stimuli [10]. This peculiar crowding property is generally referred to as ‘compulsory averaging’. In the experiment of Parkes *et al.*, observers reported the tilt direction of a variable number of equally tilted targets positioned among horizontal flankers. Parkes *et al.* found that a relatively simple pooling model could account for human data when the total number of stimuli is kept constant. However, when targets are presented without flankers, identification thresholds dropped significantly slower as a function of the number of targets than predicted by their model (Figure 5b). They postulated a ‘late noise’ factor to explain the discrepancy between data and model.

Our model suggests the following explanation for the compulsory averaging phenomenon. When two features are highly similar, their population code representations have a high degree of overlap and will merge when summed. Consequently, the resulting post-integration code will be interpreted as representing a single feature with a value somewhere in between the values of the input stimuli (Figure 5a). To examine whether our model can also quantitatively account for compulsory averaging, we conducted a simulation experiment with conditions and stimuli similar to those used in the psychophysical experiment performed by Parkes *et al.* [10]. The results show that our model produces accurate fits to the psychophysical data for both the condition with and without flankers (Figure 5b).

An important difference between our model and the pooling model proposed by Parkes *et al.* is that the latter integrates all stimuli with equal weight, while integration in our model is weighted by object spacing. To verify the relevance of this aspect in explaining why the models make different predictions, we reran the simulations with varying stimulus spacing (see Text S1 and Figure S3 for results). We found that when we set all integration weights in our model to one (implying an object spacing of zero), the identification thresholds predicted by our model are similar to those predicted by the pooling model of Parkes *et al.* Additionally, the predictions of the models increasingly diverge when object spacing is increased. These results confirm that object-spacing related weighting of integration is an essential difference between the models. Moreover, they challenge the need for the ‘late noise’ factor proposed by Parkes *et al.* to explain their results.

### Peripheral flankers cause stronger crowding than foveal flankers

Several studies [5],[29] have found that, with equal target-flanker spacing, flankers positioned at the peripheral side of a target cause stronger crowding effects than flankers positioned at the foveal side. As has been noted previously [16], this asymmetry follows directly from the way that the visual field is mapped onto the cortex. With increasing eccentricity, the representation of visual space becomes more and more compressed. Consequently, for equal target-flanker spacing in visual space, the cortical distance between the representation of a target and a foveal flanker is larger than that between a target and a peripheral flanker. Assuming that cortical integration fields are isotropic, peripheral flankers will, therefore, contribute more to the integrated target signal than foveal flankers.

**Figure 6:**
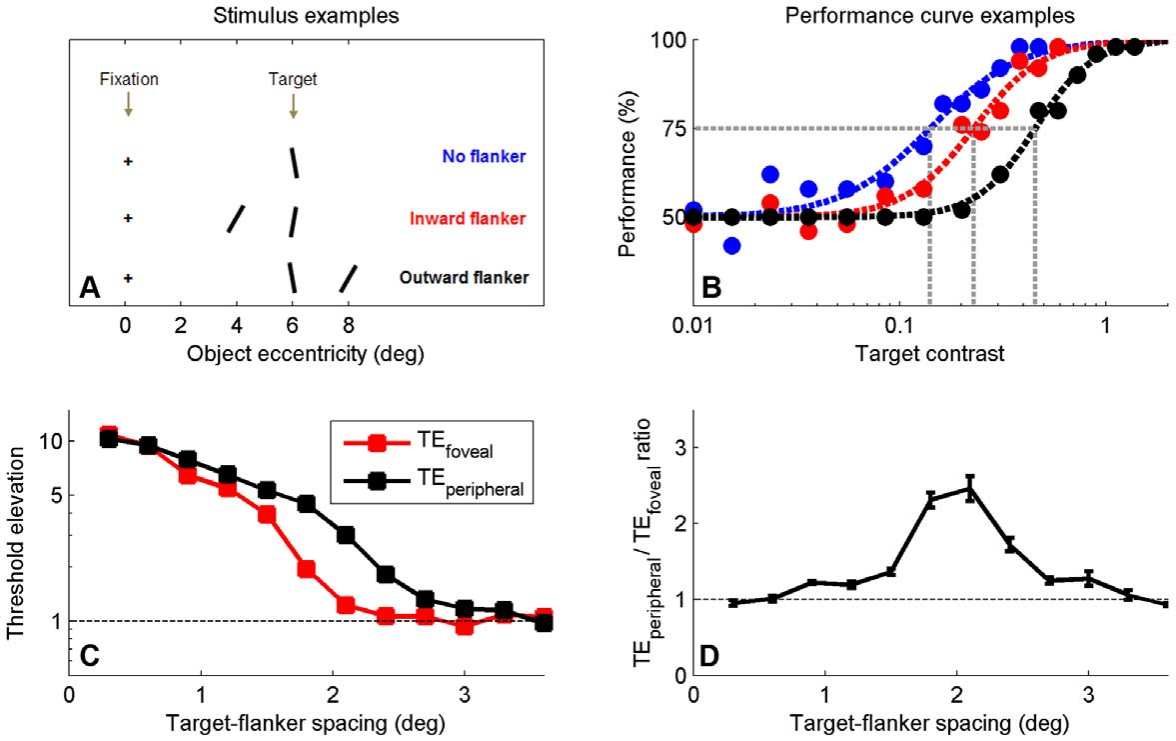
Simulation results illustrating the anisotropic effects of foveal vs peripheral flankers on target identification. A. Stimuli consisting of a ±10° tilted target, flanked by either no flanker, a foveal flanker, or a peripheral flanker. B. Both flankers elevate target tilt identification thresholds, but this effect is largest for peripheral flankers. We define threshold elevations TE_foveal_ and TE_peripheral_ as the 75%-correct target contrast found for the condition with a foveal and peripheral flanker, respectively, divided by the 75%-correct target contrast found for the condition without a flanker. C. Predicted threshold elevations plotted as a function of target-flanker spacing. When target-flanker spacing is small or when it approaches the critical spacing, the effects of foveal and peripheral flankers are comparably strong. However, in the intermediate range, a peripheral flanker produces larger threshold elevations (i.e., stronger crowding) than a foveal flanker. D. The same data as in C, but now shown as a ratio (i.e., the values at black data points from panel C divided by those at the red data points).

We conducted a simulation experiment to verify whether our model replicates the foveal-peripheral anisotropy and to investigate how its predictions depend on target-flanker spacing. For several target-flanker spacings, we estimated 75%-correct target contrast thresholds for identifying the tilt of a target without a flanker, a target with a foveal flanker, and a target with a peripheral flanker (Figure 6a). The results show that while both the foveal and peripheral flanker produce crowding (Figure 6b), the effect caused by a peripheral flanker is substantially larger than that caused by a foveal flanker (Figure 6c). Hence, our model exhibits a foveal-peripheral flanker anisotropy. Furthermore, the model predicts the anisotropy to be strongest at intermediate spacings while it predicts no anisotropy when target-flanker spacing is very small or approaches the critical spacing (Figure 6d). In these simulation data, the strongest anisotropy is found when target-flanker spacing is about 2 degrees (i.e., about 0.3 times the target eccentricity). At this spacing, threshold elevation caused by the peripheral flanker is predicted to be approximately 2.5 times that caused by the foveal flanker. This is comparable to the effect size measured for human observers [29].

### Spatial integration enhances signals from correlated stimuli

The results so far suggest that crowding is what happens when signals from closely-spaced, unrelated stimuli are integrated with each other. However, in normal viewing conditions, signals from closely-spaced stimuli are often correlated (e.g., neighboring line segments of an edge or smooth contour). It has been suggested that integration of such correlated (orientation) signals may underlie phenomena such as contour integration [25],[32],[33].

**Figure 7:**
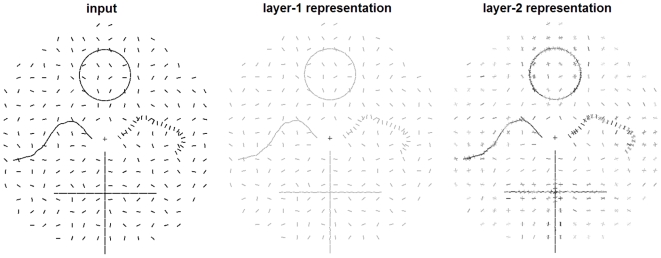
Simulation results showing how our model responds to visual contours. Left image: input stimulus, consisting of a set of oriented line segments comprising several contours within a noisy background. The ‘+’ symbol indicates the center of the visual field and was not part of the stimulus. Central image: a visualization of the stimulus representation in the first layer of our model, which is a noisy version of the input. The contrast of the bars is set to the median of the contrasts in the right image. Right image: a visualization of the decoded stimulus representations after integration. At every original input location, the post-integration population code was decoded to a mixture of normal distributions. The contrast of each bar is proportional to the associated mixing proportion. Note the highlighting of the contours and the crowding effects in the periphery, which agrees well with the subjective experience when viewing the input stimulus.

To see how our model responds to signals from correlated stimuli, we ran a simulation with an input stimulus consisting of a set of line segments comprising various contours within a noisy background (see Methods for details). The results are shown in Figure 7. Line segments that are part of a contour clearly stand out in the post-integration representation. This is because both stimulus density and orientation correlation are higher for contours than for the random background. This result supports an earlier suggested link between contour integration and crowding [22], but firm conclusions would require further extensive evaluation. Note that in areas away from fixation, in the periphery of the visual field, the decoder often returned stimulus distributions that represent more than one orientation value. This indicates that the post-integration codes at those locations are ambiguous in terms of the encoded orientation. In other words, when stimulus spacing is small relative to eccentricity, stimuli become jumbled with their neighbors, just as observed in crowding.

## Discussion

We presented a model of spatial feature integration based on the principles of population coding. While there is a growing consensus for the theory that spatial feature integration is responsible for crowding [4], the model that we presented here is the first to quantitatively account for several fundamental properties of this phenomenon in a coherent and biologically plausible manner. Besides replicating the properties of the critical spacing of crowding, and the anisotropic crowding effects of foveal versus peripheral flankers, our model also replicates and explains ‘compulsory averaging’ of crowded orientation signals. Furthermore, it suggests that crowding may be the by-product of a mechanism aimed at enhancing the saliency of ecologically relevant stimuli, such as visual contours.

### Physiological motivation

The cells in the first layer are modeled after V1 simple cells (see Methods for accompanying parameter values). However, there is currently no agreement about the cortical locus of the ‘integration cells’ that are supposed to underlie crowding. Therefore, we decided to make minimum assumptions about their physiological origin. Consequently, the size and shape of their receptive fields, determined by σ_rad_ and σ_tan_ (see Methods), were taken to be free parameters, such that the parameter values that provide a good fit to experimental data can be considered a prediction for the receptive field properties of the integration cells underlying crowding. We found that the best model fit to the data is obtained with integration cells that are strikingly similar to a type of cell that has recently been identified in V4 (of cat and monkey) [34],[35]. The function of these cells is currently unknown [36]. Hence, we speculate that these V4 cells spatially integrate information from V1 (either directly or mediated by V2). Their possible function may be contour integration (e.g., as a precursor for shape coding), with crowding as a by-product. Interestingly, other, independent, lines of evidence also have suggested that crowding occurs beyond V1 [21],[37] with V4 as a likely candidate area [38].

The parameter settings (see Methods) in our model were fixed over the entire range of simulations that we performed, with one minor exception (see Figure 3). We reran a number of simulations with different parameter values and found that this hardly affected our results (see Text S1 and Figure S4 for details). This suggests that crowding is an inherent property of a mechanism that integrates signals by summing population codes.

### Comparison with other theories

These results shed new light on earlier proposed crowding theories. Some authors have proposed that crowding is, at least in part, the result of ‘source confusion’ due to positional uncertainty [39],[40]. We would like to note, however, that integrating signals over space necessarily increases positional uncertainty. Hence, we consider location uncertainty and, consequently, ‘source confusion’ a result of feature integration, rather than an additional factor in the explanation of crowding. Indeed, our results show clear evidence for ‘source confusion’, even though we did not explicitly incorporate positional uncertainty into our model (for an example, see Figure 5a).

When spatially averaging signals in a retinotopically arranged ‘feature map’ (such as V1), activation patterns that are caused by closely spaced stimuli may slightly shift towards each other (or even completely merge together, if spacing is very small). As a result, an averaging of stimulus positions may be perceived in such situations. In a recent paper it was shown that judgments of the position of a crowded target object are systematically biased towards the positions of flanking objects [41]. The authors of that paper explained their results by a model that averages stimulus positions. Based on the foregoing argument, their results can presumably just as well be explained as a result of averaging feature signals over space.

A recent theory suggests that crowding is the ‘breakdown of object recognition’ [3]. The reasoning is that spatial integration of object features (in the notion of ‘binding’) is required for object recognition, whereas crowding occurs when multiple objects fall within the same integration field. Our results indicate that the spatial signal integration underlying crowding may enhance responses for correlated signals, such as contours. This corroborates an earlier suggestion that the ‘association fields’ that have been proposed to underlie contour integration [42] may also cause crowding [22]. While such enhancement of responses to correlated signals will no doubt facilitate higher-order functions such as object recognition, integration appears to have a more elementary and general function.

Other authors argue that crowding is the result of attentional limitations [20],[21], although evidence for these theories is considered very slim [4]. While we deem it possible that attentional factors have modulatory effects on crowding, our present results show that the general properties of crowding can very well be accounted for without invoking attentional mechanisms.

It has also been suggested that crowding is ‘texture perception when we do not wish it to occur’ [10]. The motivation behind this proposal is the finding that observers cannot identify individual stimulus properties in a crowded display, but still have access to its average statistics (i.e., its texture properties). Our model is able to explain this finding (see Figure 5), and we agree that what occurs after pooling can be described as ‘texture perception’. However, in view of the plausible connection between spatial integration and contour integration, we hesitate to conclude that texture perception is the primary function of spatial integration. Moreover, if a functional link exists between spatial integration and texture perception, then we deem it just as likely that integration serves to compress visual information, in order to reduce energy requirements at higher levels of processing.

### Limitations

Two crowding properties that our current model does not account for are the effects of ‘target-flanker similarity’ and ‘flanker configuration’. The former refers to the finding that crowding is stronger for target-like flankers compared to dissimilar flankers [9],[43],[44]. The ‘flanker configuration’ effect refers to the finding that crowding is partially ‘released’ when surrounding flankers form a contour [45],[46]. A rather natural extension to our model may allow it to account for these two effects as well. At present, the integration fields in our model represent exclusively excitatory horizontal connections between cells. Alongside these excitatory connections, however, many of the cells in primary visual cortex are known to have inhibitory connections as well as feedback connections from higher-order brain areas [47]. Inhibition could reduce the integration of dissimilar pieces of information and thus be responsible for target-flanker similarity effects in crowding. Likewise, the feedback connections might inhibit the integration of signals that are likely to represent different objects or ‘perceptual groups’ and, therefore, be responsible for configuration influences on crowding.

### Generalization to crowding in other domains

The model and simulations that were presented in this paper are limited to the orientation domain. However, crowding is a rather general phenomenon that affects a large number of tasks, including discrimination of letters and objects sizes, colors, and shapes. Since population coding is considered the general way by which variables are encoded in the brain [24], crowding of other basic features such as size and color [12] can presumably be explained by a model that is largely analogous to the one presented here. Moreover, if population coding is also used to encode more complex information, and spatial integration takes place at many different levels of processing, then our model predicts that crowding should also be found at many different levels. Hence, crowding of more complex structures (such as letters, object shapes, bodies, and faces) could follow both from crowding in their constituent features and from crowding within higher-order population codes that represent the structures themselves [48].

### Predictions

Our model licenses a number of predictions that can be tested experimentally. For example, the simulations related to the ‘compulsory averaging’ effect predicts the effects of stimulus spacing and contrast on identification thresholds. Additionally, the model makes quantitative predictions regarding the effect of spacing on the foveal-peripheral flanker anisotropy of crowding. Finally, the model makes predictions about the receptive field properties of the integration cells responsible for crowding,

### Conclusion

The results that we presented here lend strong quantitative support to the theory that the mechanism behind crowding is spatial feature integration, and our model provides a computationally motivated physiological basis to this theory.

## Methods

### Model

Input stimuli are specified as 4-tuples 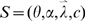, where 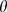 is the orientation, 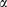 the size, 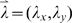 the location, and 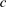 the (relative) contrast of the stimulus. For each of these inputs we first define a corresponding probability distribution, which is subsequently used as input to the distributional population coding scheme of Zemel *et al.* [31]. The width 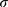 of an input distribution represents the perceptual uncertainty about a stimulus and is related to stimulus eccentricity 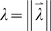, size 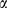, and contrast *c*, in the following way (see Text S1 for motivation):
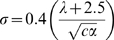
(1)In order to account for the circularity of the orientation domain, we define these distributions to be circular normal (von Mises) distributions. More specifically, the distribution over orientation *s* for a stimulus 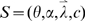 is defined as:
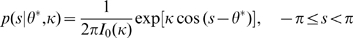
(2)where 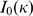 is the modified Bessel function of order 0, 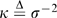 is an inverse measure of statistical dispersion, and 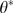 is a value drawn from the normal distribution 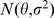 over *s*. In the simulation experiments we map the stimulus domain [−90,90) deg to [−π, π). The tuning curves *f_i_(s)* of the cells are defined as circular normal functions over *s*:
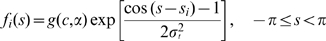
(3)where *s_i_* is the preferred orientation of cell *i*, 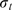 the width of the tuning curves, and 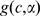 an S-shaped function that defines how cell gain relates to the contrast *c* and size *α* of a stimulus (see Text S1 and Figure S1).

Following the DPC scheme, we compute the average response of cell *i* to a stimulus 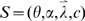 as follows:
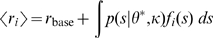
(4)where 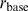 is the level of spontaneous activity and 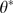 drawn from a normal distribution with mean 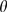 and a standard deviation 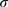. In order to evaluate this integral numerically, we approximate the input distributions 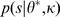 by histograms 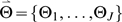 and the tuning functions 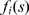 by histograms 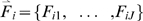, both with bin centres linearly spaced in the range 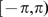. Hence, we can rewrite equation (4) to
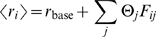
(5)A population code 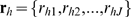 representing a stimulus 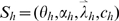 is constructed by drawing responses *r_hi_* from Poisson distributions
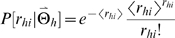
(6)


The second layer of the model spatially integrates the stimulus representations in the first layer. The layer-2 population code 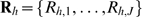 that is associated with position 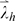 is computed as a weighted sum over the population code representations of all *N* input stimuli:
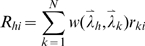
(7)where 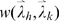 is a 2D Gaussian weight function that represents the cortical integration fields (see Text S1 and Figure S2 for details).

Several of our simulation experiments require that a task response is generated. In those experiments, a Bayesian decoder is used to estimate the stimulus probability distribution that is encoded in the post-integration population code associated with the target position. Subsequently, the orientation with the highest probability is interpreted as representing the most likely orientation of the target, and chosen for response. We use the Bayesian Information Criterion to choose the most likely mixture model among a set of models with 1, 2, and 3 mixture components. We refer to the Text S1for all mathematical details of the decoder.

### Model parameters

The parameter settings of the model were as follows. In all simulations, the width of the tuning curves 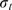 was set to 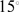, the number of neurons *J* comprising one population code was set to 90, the spontaneous firing rate *r*_base_ was set to 5 spikes/s, and the maximum firing rate was set to 90 spikes/s. The only parameters that varied between simulations were σ_rad_ and σ_tan_, which determine the integration field width in the ‘radial’ and ‘tangential’ direction, respectively (see Text S1). These were set to 2.5 and 1.0 mm, respectively, in all simulations, except the one in which we estimated critical regions (Figure 3), where the values were set to 1.6 and 1.1mm, respectively. This difference is motivated by the observation that the human data in Figure 3 are from a subject with an unusually small critical spacing (approximately 0.3 times the target eccentricity).

### Estimation of target identification thresholds and critical spacing

Several simulation experiments involved estimation of target contrast thresholds for a tilt identification task. In those experiments, the procedure on a single trial was as follows. The target and flanker stimuli were encoded and their representations integrated, as described above. Subsequently, the post-integration population code associated with the target position was decoded to a mixture of normal distributions. The sign of the orientation associated with the peak location in the returned probability distribution was compared with the sign of the input target. Performance was considered ‘correct’ if the signs were the same, and ‘incorrect’ otherwise. Performance estimates were made for several target contrasts, by simulating 50 trials for each contrast. Finally, a sigmoid function with a mean *a* and a width *b*:
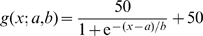
(8)was fit to these data, in order to obtain an estimate of the target contrast that yields 75%-correct performance (see Figure 3b for an example).

In the simulation experiments that estimated critical spacing, the above procedure was repeated to obtain 75%-correct thresholds for several target-flanker spacings. A ‘clipped line’ was fit to these thresholds in order to estimate critical spacing (see Figure 3c for an example).

### Estimation of critical regions (Figure 3)

Input stimuli consisted of a ±10° tilted target and two 30° tilted flankers, positioned at opposite sides of the target. Flanker contrast and the size of both the target and flankers were set to 1. Using the procedure described above, critical spacing was estimated for the same target and flanker positions as in the psychophysical experiment by Pelli *et al.* [15].

### Effect of stimulus properties on critical spacing (Figure 4)

The input stimuli consisted of a ±10° tilted target, one −30° tilted flanker, and one +30° tilted flanker. Flanker contrast and the size of both the target and flankers were set to 1. Critical spacing was determined for flankers positioned along the radial axis, on opposite sides of the target.

### Compulsory averaging of crowded orientation signals (Figure 5)

In the first simulation (Figure 5a), input stimuli consisted of a 0° tilted target and two flankers with 10° tilt in the first condition and 50° tilt in the second condition. The target was positioned at 2.5 deg of eccentricity. The flankers were positioned on opposite sides of the target, with a spacing of 0.5 deg of eccentricity. The contrast and size of all stimuli were set to 1. Stimuli used in the second simulation (Figure 5b) were similar to those used in the psychophysical experiment by Parkes *et al.* [10]: N tilted targets and 9-N vertical flankers (first condition) or no vertical flankers (second condition), with a central position of 2.5 deg of eccentricity and a spacing of 0.5 deg between the central stimulus and surrounding stimuli. The contrast and size of the stimuli were set to 0.5. On a single trial, the post-integration population code associated with the central stimulus position was decoded to a unimodal stimulus distribution. The sign of the orientation with the highest probability was compared with the sign of the target. If they were the same, performance on that trial was considered correct. We measured performance over 100 trials for varying target tilts. Based on these data, 75%-correct performance thresholds were determined. This procedure was repeated for different values of N.

### Foveal-peripheral flanker anisotropy (Figure 6)

Input stimuli consisted of a ±10° tilted target without a flanker (condition 1), with a 30° tilted foveal flanker (condition 2), or a 30° tilted foveal flanker (condition 3). Flanker contrast and the size of both the target and flankers were set to 1. For all three conditions, 75%-correct target contrasts were estimated for a range of target-flanker spacings. Threshold elevations *TE_foveal_* and *TE_peripheral_* were defined as described in the main text.

### Model response to visual contours (Figure 7)

The input stimuli consisted of a set of oriented bars, comprising three contours within a field of randomly oriented bars. The circle contour consisted of 35 equally spaced segments, was centered at (0,10) degrees of eccentricity and had a radius of 4 degrees of visual angle. The other four contours consisted of 23 line segments each, with a spacing of 0.7 degrees of visual angle between every two neighboring segments. The randomly oriented line segments were placed on a grid with a radius of 18 degrees of eccentricity and a grid spacing of 2.0 degrees. The contrast and size of all line segments was set to 0.8.

## Supporting Information

Figure S1 Graphical illustration of the function used in the model to relate the response gain of a population code to the (relative) size and contrast of the stimulus that it encodes.
(0.08 MB TIF)

Figure S2 A graphical illustration of how the ‘radial’ and ‘tangential’ distance between an integration field and stimulus are computed. A. Visualization of the right visual hemifield. The red marker indicates the center location of an integration field. The blue marker indicates the location of a stimulus. B. Cortical representation of the visual hemifield. C. The cortical distance between the integration field center and the stimulus along the eccentricity axis is defined as the ‘radial’ distance. The distance along the orthogonal axis is defined as the ‘tangential’ distance.
(0.31 MB TIF)

Figure S3 Predicted identification thresholds for a target identification task with N equally tilted targets and no flankers. Thresholds predicted by our model depend on object spacing. For a spacing of 0, the predictions match those from the pooling model by Parkes et al.; for a spacing of 0.5, the predictions of our model match the psychophysical data that were measured with the same object spacing; for spacings that are close to or larger than the critical spacing, our model predicts that identification thresholds are independent of the number of targets. Human data from [4], subject LP.
(0.15 MB TIF)

Figure S4 Results of a simulation that estimated critical spacing for a tilt identification task of a target located at 6 degrees of eccentricity. The stimuli and procedure were the same as for the simulations in the main experiment. These results show that critical spacing is hardly affected by the model parameters, which indicates that critical spacing is a general property of the type of model that we proposed.
(0.45 MB TIF)

Text S1 Mathematical details of the model described in the main text, and supplementary simulation results.
(0.19 MB DOC)
