# Supplementary figures and images for: A Neurophysiologically Plausible Population Code Model for Feature Integration Explains Visual Crowding
Source: PLoS Comput Biol. 2010 Jan 22;6(1):e1000646. doi: 10.1371/journal.pcbi.1000646 (PMC2799670; doi:10.1371/journal.pcbi.1000646)

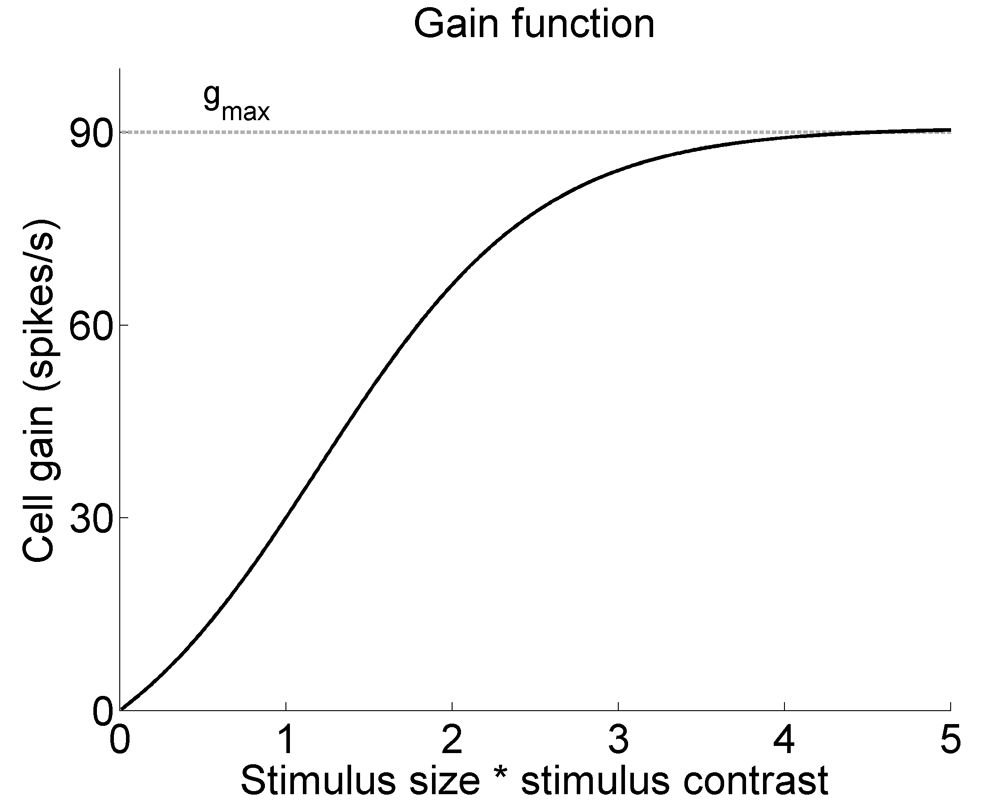

Supplement: Figure S1 — Graphical illustration of the function used in the model to relate the response gain of a population code to the (relative) size and contrast of the stimulus that it encodes. (0.08 MB TIF) [file pcbi.1000646.s001.tif]

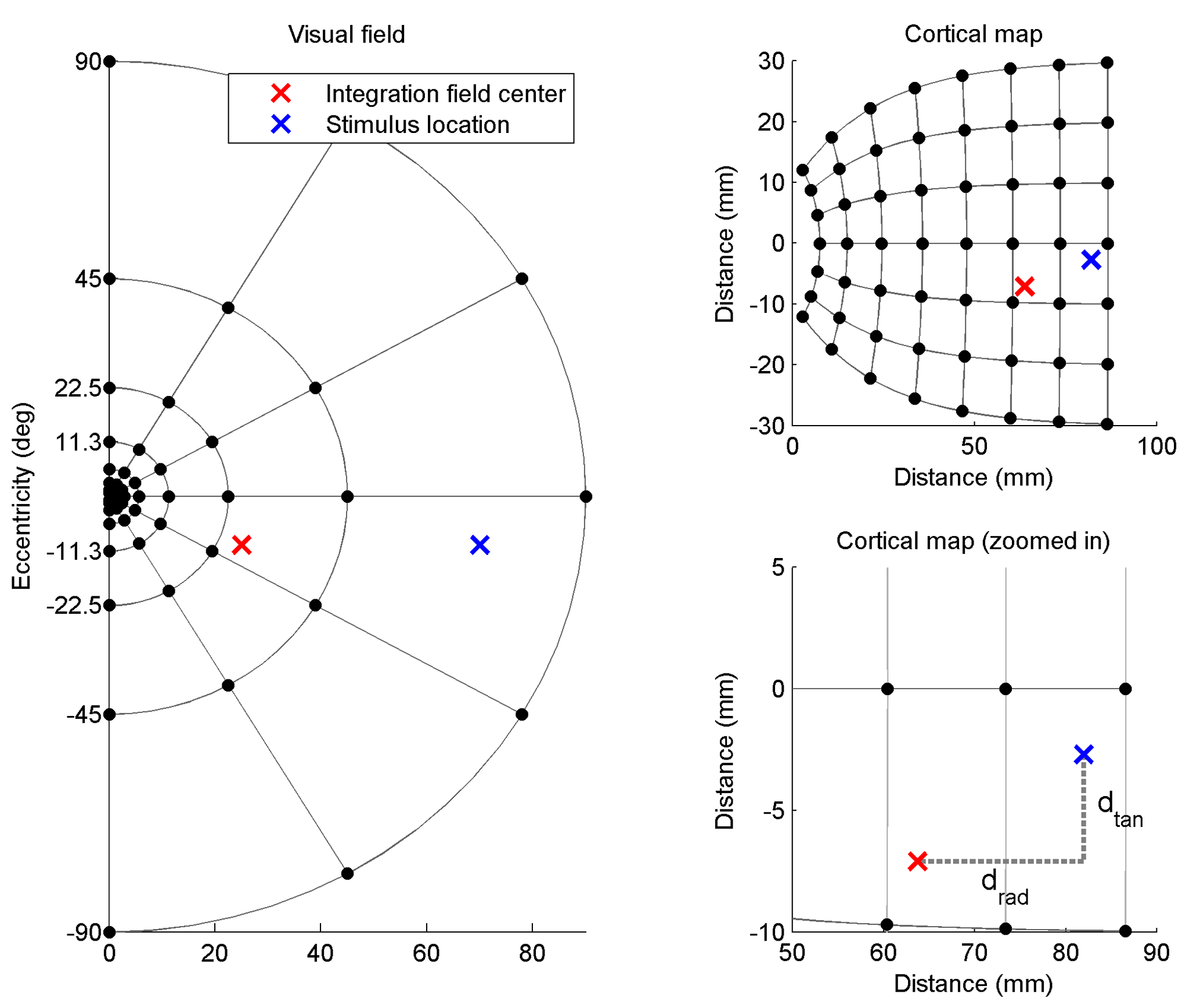

Supplement: Figure S2 — A graphical illustration of how the ‘radial’ and ‘tangential’ distance between an integration field and stimulus are computed. A. Visualization of the right visual hemifield. The red marker indicates the center location of an integration field. The blue marker indicates the location of a stimulus. B. Cortical representation of the visual hemifield. C. The cortical distance between the integration field center and the stimulus along the eccentricity axis is defined as the ‘radial’ distance. The distance along the orthogonal axis is defined as the ‘tangential’ distance. (0.31 MB TIF) [file pcbi.1000646.s002.tif]

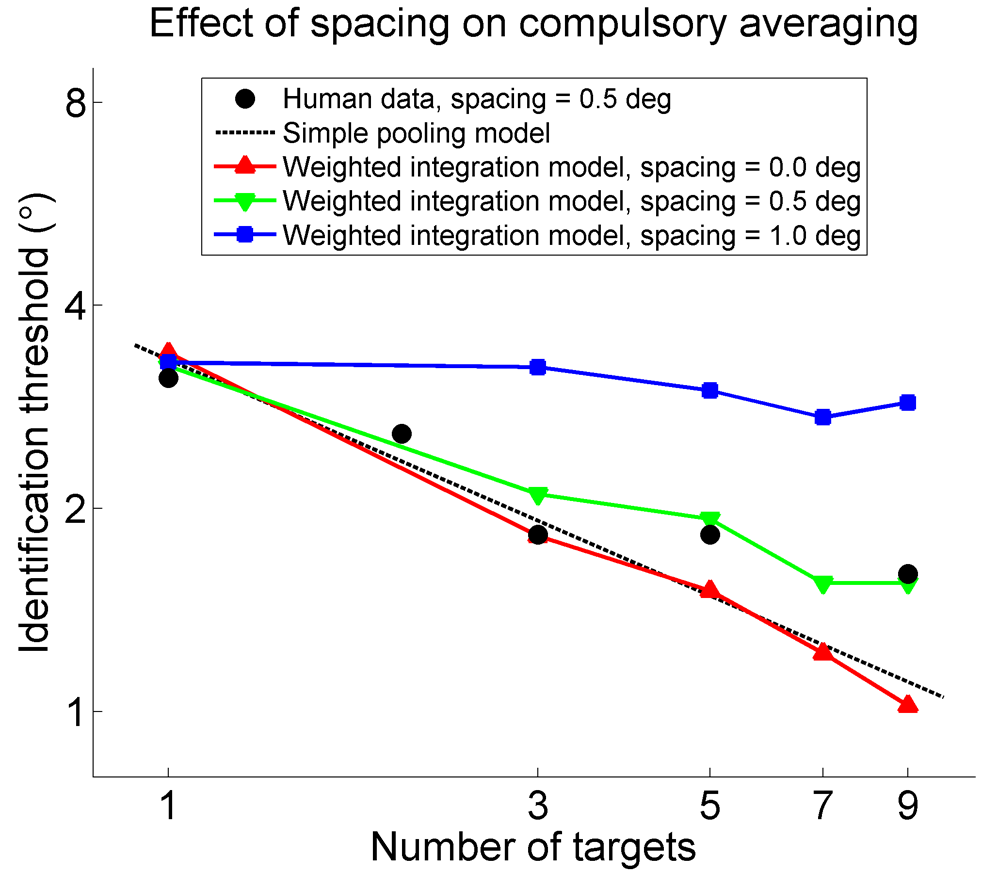

Supplement: Figure S3 — Predicted identification thresholds for a target identification task with N equally tilted targets and no flankers. Thresholds predicted by our model depend on object spacing. For a spacing of 0, the predictions match those from the pooling model by Parkes et al.; for a spacing of 0.5, the predictions of our model match the psychophysical data that were measured with the same object spacing; for spacings that are close to or larger than the critical spacing, our model predicts that identification thresholds are independent of the number of targets. Human data from [4], subject LP. (0.15 MB TIF) [file pcbi.1000646.s003.tif]

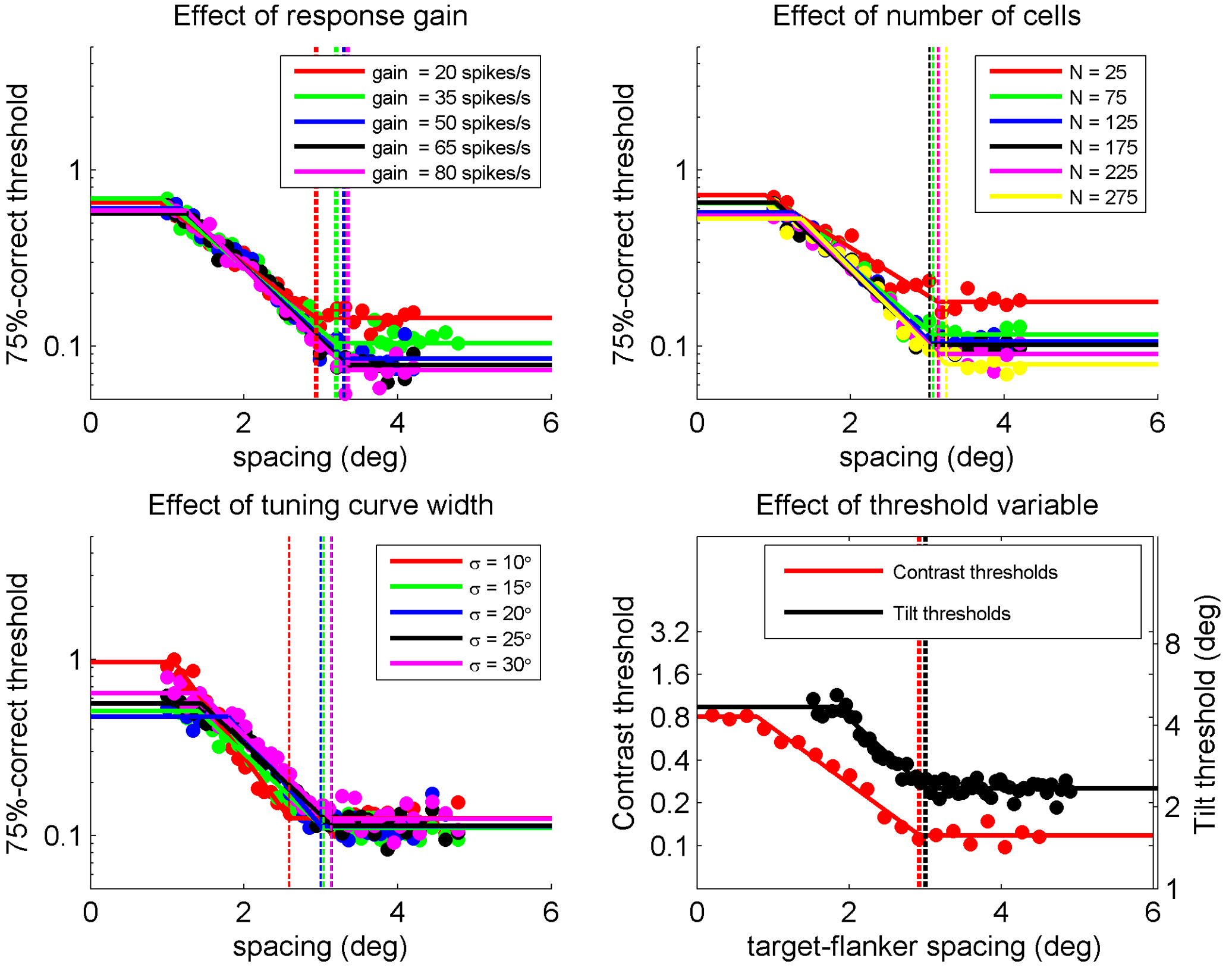

Supplement: Figure S4 — Results of a simulation that estimated critical spacing for a tilt identification task of a target located at 6 degrees of eccentricity. The stimuli and procedure were the same as for the simulations in the main experiment. These results show that critical spacing is hardly affected by the model parameters, which indicates that critical spacing is a general property of the type of model that we proposed. (0.45 MB TIF) [file pcbi.1000646.s004.tif]
