# Supplementary material for: A Neurophysiologically Plausible Population Code Model for Feature Integration Explains Visual Crowding
Source: PLoS Comput Biol. 2010 Jan 22;6(1):e1000646. doi: 10.1371/journal.pcbi.1000646 (PMC2799670; doi:10.1371/journal.pcbi.1000646)
Supplement: Text S1 — Mathematical details of the model described in the main text, and supplementary simulation results. (0.19 MB DOC) [file pcbi.1000646.s005.doc]

**Supplementary information**

This document contains Supplementary Materials for *A neurophysiologically plausible population code model for feature integration explains visual crowding* (R. Van den Berg, J.B.T.M. Roerdink, & F.W. Cornelissen, 2010). It consists of two parts: Supplementary Methods, which provides mathematical details and additional information about the model, and Supplementary Results, which provides additional simulation results for which we did not have space in the main text.

**SUPPLEMENTARY METHODS**

**Stimulus encoding**

Input stimuli are specified as 4-tuples $S=(\theta,\alpha,\vec{\lambda},c)$, where $\theta$ is the orientation*,* $\alpha$the size, $\vec{\lambda}=(\lambda_{x},\lambda_{y})$ the location, and $c$the (relative) contrast of the stimulus. In the first layer of the model, the distributional population coding (DPC) scheme by Zemel *et al.* [1] is used to compute internal population code representations for these stimuli. We first define probability distributions for the input stimuli, which capture the stimulus uncertainty caused by neural noise in processing stages prior to the first layer of the model. We relate the width $\sigma$of these distributions to the eccentricity$\lambda=\left\|\vec{\lambda}\right\|$, size $\alpha$, and contrast *c* of a stimulus, in the following way. From [2] we know that the cortical magnification factor $M(\lambda)$ - which describes the differential change in cortical position with respect to retinal eccentricity$\lambda$ - can be approximated as follows:

$M(\lambda)\approx\frac{8}{0.4\lambda+1}$ (S1)

From this, we infer that the cortical size *A* of a stimulus that subtends $\alpha$deg^2^ of visual angle and which is centered at $\lambda$ degrees of eccentricity, can be approximated as follows:

$A(\alpha,\lambda)\approx\alpha\left[M(\lambda)\right]^{2}\approx\frac{64\alpha}{\left(0.4\lambda+1\right)^{2}}$ (S2)

Assuming that the uncertainty about the value of a stimulus is inversely proportional to both the size of its cortical representation and its contrast, we obtain the following relation between stimulus uncertainty $\sigma^{2}$ on the one hand, and stimulus eccentricity$\lambda$, contrast *c*, and size $\alpha$on the other hand:

$\begin{matrix} \sigma^{2}\propto\frac{1}{A(\alpha,\lambda)c}\approx\frac{\left(0.4\lambda+1\right)^{2}}{64\alpha c} \\ \sigma\propto\frac{1}{\sqrt{A(\alpha,\lambda)c}}\approx\frac{0.4\lambda+1}{8\sqrt{\alpha c}}=\frac{0.4(\lambda+2.5)}{8\sqrt{\alpha c}}\propto\frac{\lambda+2.5}{\sqrt{\alpha c}} \end{matrix}$ (S3)

In our model, we choose a constant of proportionality of 0.4:

$\sigma=0.4\left(\frac{\lambda+2.5}{\sqrt{\alpha c}}\right)$, (S4)

which gives, for example, $\sigma=1$deg for a foveally presented stimulus with a size of 1 deg^2^ and a contrast of 1, and $\sigma=4$for the same stimulus at 7.5 degrees of eccentricity.

We define the gain of a population code by an S-shaped function of stimulus contrast *c* and size *α*:

$g(c,\alpha)=g_{\max}\left(1-\frac{q}{1+e^{t}}\right)$ (S5)

where *g*_max_ is the maximum firing rate of a cell, *q*=1.16 is a constant, and $t=a(c\alpha-b)$, with constants *a=*1.5 and *b*=1.2 (see Figure S1 for a plot of this function). Note that stimulus contrast and size have the same effect on response gain.

**Weight function**

The integration weights in our model depend on the distance between the (center of an) integration field and the stimulus positions. Since these distances are computed in ‘cortical space’, we first compute the cortical locations (in primary visual cortex) of both the integration field center and the stimuli. For this, we use the complex log mapping from [3] to map visual field locations $\vec{\lambda}$to cortical locations:

, (S6)

where *E* and *φ* are the eccentricity and angle of the polar representation of $\vec{\lambda}$, *k*=19.2, *a*=0.77 are constants, and is a shear function that is approximated by

, (S7)

where S_1_=0.76 and S_2_=0.18 are constants.

The weight function that determines how strongly the response of a cell that encodes a stimulus at position $\vec{\lambda}_{h}$ contributes to the response of an ‘integration cell’ associated with position $\vec{\lambda}_{k}$, is defined by a 2D Gaussian function of cortical distance between both cells:

$w(\vec{\lambda}_{h},\vec{\lambda}_{k})=\exp\left(-\frac{\left[\text{d}_{\text{rad}}(\vec{\lambda}_{h},\vec{\lambda}_{k})\right]^{2}}{2\sigma_{\text{rad}}^{2}}-\frac{\left[\text{d}_{\text{tan}}(\vec{\lambda}_{h},\vec{\lambda}_{k})\right]^{2}}{2\sigma_{\text{tan}}^{2}}\right)$, (S8)

where $\text{d}_{\text{rad}}(\vec{\lambda}_{h},\vec{\lambda}_{k})$ and $\text{d}_{\tan}(\vec{\lambda}_{h},\vec{\lambda}_{k})$ give the ‘radial’ and ‘tangential distance’ between the cortical locations of the cells, and $\sigma_{\text{rad}}$ and $\sigma_{\text{tan}}$ determine the size of the integration field in the ‘radial’ and ‘tangential direction’, respectively (see Figure S2 for computation of ‘radial’ and ‘tangential’ distances).

**Signal decoding**

Several of our simulation experiments require that a task response is generated. In those experiments, a Bayesian decoder is used to estimate the stimulus probability distribution $\vec{\Psi}$that is encoded in the post-integration population code **R** associated with the target position. Subsequently, the orientation with the highest probability is interpreted as representing the most likely orientation of the target, and chosen for response. We assume that the distribution encoded in a post-integration population code is a mixture model of *K* von Mises distributions, which we approximate by a histogram$\vec{\Psi}=\sum_{k=1}^{K} \Psi_{k}$, with each component $\Psi_{k}=\{\Psi_{k1},\text{ }\ldots\text{ },\Psi_{kJ}\}$ specified as follows:

$\Psi_{kj}=\frac{p_{k}}{2\pi I_{0}(\kappa_{_{k}})}\exp\left[\kappa_{_{k}}\cos(s_{j}-\mu_{k})\right]$, (S9)

where $I_{0}(\kappa)$ is the modified Bessel function of order 0, $\kappa\triangleq\sigma^{-2}$ is an inverse measure of statistical dispersion, $s_{j}$ is the orientation corresponding to the *j*-th bin of the histograms, and$p_{k}$ are the mixing proportions, $\mu_{k}$the means, and $\sigma_{k}^{2}$ the variances of the mixture components.

Assuming statistical independence of cell responses, applying Bayes’ rule and assuming a flat prior over$\vec{\Psi}$, we obtain the following likelihood function:

$\begin{matrix} P[\vec{\Psi}|\vec{R}]\propto P[\vec{R}|\vec{\Psi}]=\prod_{i=1}^{N} e^{-\left\langle R_{i}\right\rangle}\frac{\left\langle R_{i}\right\rangle^{R_{i}}}{r_{i}!} \\ \propto\prod_{i=1}^{N} \exp\left[-r_{\text{base}}-\sum_{jk} \Psi_{kj}F_{ij}\right]\left[r_{\text{base}}+\sum_{jk} \Psi_{kj}F_{ij}\right]^{R_{i}} \\ =\exp\left[-Nr_{\text{base}}-\sum_{ijk} \Psi_{kj}F_{ij}\right]\prod_{i} \left[r_{\text{base}}+\sum_{jk} \Psi_{kj}F_{ij}\right]^{R_{i}} \end{matrix}$ (S10)

Taking logs, we find:

$\begin{matrix} \log\left(P[\vec{\Psi}|\vec{R}]\right)=\log\left(\exp\left[-Nr_{\text{base}}-\sum_{ijk} \Psi_{kj}F_{ij}\right]\prod_{i} \left[r_{\text{base}}+\sum_{jk} \Psi_{kj}F_{ij}\right]^{R_{i}}\right)+C \\ =\sum_{i} R_{i}\log\left[r_{\text{base}}+\sum_{jk} \Psi_{kj}F_{ij}\right]-Nr_{\text{base}}-\sum_{ijk} \Psi_{kj}F_{ij}+C \end{matrix}$ (S11)

where *C* is a constant. The maximum likelihood parameters were estimated by using a gradient descent method. The partial derivatives of the log likelihood function are as follows:

$\frac{\partial\log\left(P[\vec{R}|\Psi]\right)}{\partial p_{q}}=\sum_{i} R_{i}\frac{\sum_{j} \frac{1}{p_{q}}\Psi_{qj}F_{ij}}{r_{\text{base}}+\sum_{jk} \Psi_{kj}F_{ij}}-\sum_{ij} \frac{1}{p_{q}}\Psi_{qj}F_{ij}$ (S12)

$\begin{matrix} \\ \frac{\partial\log\left(P[\vec{R}|\vec{\Psi}]\right)}{\partial\mu_{q}}=\sum_{i} R_{i}\frac{\sum_{j} \kappa_{q}\sin(s_{j}-\mu_{q})\Psi_{qj}F_{ij}}{r_{\text{base}}+\sum_{jk} \Psi_{kj}F_{ij}}-\sum_{ij} \kappa_{q}\sin(s_{j}-\mu_{q})\Psi_{qj}F_{ij} \end{matrix}$ (S13)

$\begin{matrix} \frac{\partial\log\left(P[\vec{R}|\vec{\Psi}]\right)}{\partial\kappa_{q}}=\sum_{i} R_{i}\frac{\sum_{j} \left(\cos(s_{j}-\mu_{q})-\frac{I_{1}(\kappa_{q})}{I_{0}(\kappa_{q})}\right)\Psi_{qj}F_{ij}}{r_{\text{base}}+\sum_{jk} \Psi_{kj}F_{ij}} \\ -\sum_{ij} \left(\cos(s_{j}-\mu_{q})-\frac{I_{1}(\kappa_{q})}{I_{0}(\kappa_{q})}\right)\Psi_{qj}F_{ij} \end{matrix}$ (S14)

where $I_{1}(\kappa_{q})$ is the first derivative of $I_{0}(\kappa_{q})$with respect to $\kappa_{q}$.

Determining the most likely number of stimuli encoded in a population code is an open problem in the theoretical neurosciences. Therefore, here we simply assume that in the brain a mechanism exists to estimate this number, without concerning ourselves with the neural implementation of this mechanism. We approximate the most likely number of stimuli represented in a population code by decoding to mixture distributions with 1, 2, and 3 components, and computing the Bayesian Information Criterion (BIC) for each of these mixtures:

$\text{BIC}=-2\log L+k\log J$ (S15)

where *L* is the likelihood value for an estimated model, *k* the number of parameters of the associated model, and *J* the number of data points (i.e., the length of the population code). The mixture model with the lowest BIC was chosen as the most likely stimulus distribution encoded by the respective population code.

**SUPPLEMENTARY RESULTS**

**Effect of object spacing on compulsory averaging predictions**

In the main text of this paper, we showed that our model successfully replicates the effect of ‘compulsory averaging’ of crowded orientation signals that was reported in [4]. The plot in Figure S3 shows how object spacing affects identification thresholds predicted by our model. A number of interesting observations can be made from this figure. First, when spacing is set to 0.5, our model provides a good fit to the human data that were found with the same object spacing. Second, when spacing is set to 0, the predictions of our model are the same as the predictions of the pooling model proposed by Parkes *et al.* Finally, when spacing is set to a value close to or larger than the critical spacing (which was about 1.25 deg in this simulation), then predicted identification thresholds are independent of the number of targets, as one would expect.

**Effect of model parameter settings on our main results**

We reran the simulation experiment that was used to estimate critical spacings (see main text) with a range of different parameter settings. The results are shown in Figure S4. We observe that the critical spacing predictions are hardly affected by the parameter settings, indicating that critical spacing is a rather general property of the population code integration model that we presented. Note that the floor thresholds increase when we reduce the gain or the number of neurons in the model. This is to be expected, because lower gain or fewer neurons means that there is less information (spikes) in the population codes, which will increase stimulus uncertainty.

**REFERENECS**

1. Zemel RS, Dayan P, Pouget A (1998) Probabilistic interpretation of population codes. Neural Comput 10: 403-430.

2. Rovamo J, Virsu V (1979) An estimation and application of the human cortical magnification factor. Exp Brain Res 37: 495-510.

3. Schira MM, Wade AR, Tyler CW (2007) Two-dimensional mapping of the central and parafoveal visual field to human visual cortex. J Neurophysiol 97: 4284-4295.

4. Parkes L, Lund J, Angelucci A, Solomon JA, Morgan M (2001) Compulsory averaging of crowded orientation signals in human vision. Nat Neurosci 4: 739-744.

**FIGURE LEGENDS**

Figure S1. Graphical illustration of the function used in the model to relate the response gain of a population code to the (relative) size and contrast of the stimulus that it encodes.

Figure S2. A graphical illustration of how the ‘radial’ and ‘tangential’ distance between an integration field and stimulus are computed. A. Visualization of the right visual hemifield. The red marker indicates the center location of an integration field. The blue marker indicates the location of a stimulus. B. Cortical representation of the visual hemifield. C. The cortical distance between the integration field center and the stimulus along the eccentricity axis is defined as the ‘radial’ distance. The distance along the orthogonal axis is defined as the ‘tangential’ distance.

Figure S3. Predicted identification thresholds for a target identification task with N equally tilted targets and no flankers. Thresholds predicted by our model depend on object spacing. For a spacing of 0, the predictions match those from the pooling model by Parkes et al.; for a spacing of 0.5, the predictions of our model match the psychophysical data that were measured with the same object spacing; for spacings that are close to or larger than the critical spacing, our model predicts that identification thresholds are independent of the number of targets. Human data from [4], subject LP.

Figure S4. Results of a simulation that estimated critical spacing for a tilt identification task of a target located at 6 degrees of eccentricity. The stimuli and procedure were the same as for the simulations in the main experiment. These results show that critical spacing is hardly affected by the model parameters, which indicates that critical spacing is a general property of the type of model that we proposed.
